# Effects of Elevated Temperatures on the Properties of Cement Mortars with the Iron Oxides Concentrate

**DOI:** 10.3390/ma14010148

**Published:** 2020-12-31

**Authors:** Jolanta Borucka-Lipska, Piotr Brzozowski, Jarosław Błyszko, Roman Bednarek, Elżbieta Horszczaruk

**Affiliations:** 1 Department of Reinforced Concrete Structures and Concrete Technology, Faculty of Civil and Environmental Engineering, West Pomeranian University of Technology in Szczecin, 70-310 Szczecin, Poland; jolanta.borucka-lipska@zut.edu.pl (J.B.-L.); piotr.brzozowski@zut.edu.pl (P.B.); jaroslaw.blyszko@zut.edu.pl (J.B.); 2 Department of Geotechnics, Faculty of Construction and Environmental Engineering, West Pomeranian University of Technology in Szczecin, 70-310 Szczecin, Poland; roman.bednarek@zut.edu.pl

**Keywords:** iron oxides concentrate, cement mortars, high temperature, mechanical performance

## Abstract

Using the waste materials in the production of the building materials limits the storage of the wastes, burdensome for the environment and landscape, and makes possible to manufacture the materials and products with the use of the less volume of the raw materials. Cement concretes and mortars as the basic building materials offer the broad prospects of utilization of the recyclable or waste materials. The wastes from the iron ore processing are the solid wastes resulting from the process of enrichment of the ore concentrate. The paper presents the results of testing three mortars, in which a part of fine aggregate was replaced with the iron oxide concentrate (IOC) resulting from such a process. IOC has been used as a substitute of 10%, 20% and 30% (by mass) of the fine aggregate. The effect of the concentrate on the mechanical performance of the mortars at the high temperature (up to 600 °C) was also investigated. The IOC is a neutral material, not affecting chemically the process of cement hydration. The addition of IOC slightly improves the strength of the cement mortars (by 5% to 10%). In the case of the larger amount (20–30%) of the addition, the use of superplasticizer is necessary. The IOC significantly improves the high temperature resistance of the cement mortars (300 °C). The cement mortars containing 30% of the IOC addition keep 80% of the initial flexural and compressive strength when exposed to the temperature 450 °C.

## 1. Introduction

One of the goals in the sustainable construction is limitation of the use of the natural raw materials towards the utilization of the recyclable or waste materials. The use of the wastes diminishes the need for their storage, burdensome for the environment and landscape, as well as makes possible to manufacture the materials and products with the use of the less volume of the primary raw materials. Cement concretes and mortars as the basic building materials offer the broad prospects of utilization of the recyclable or waste materials [1,2,3,4].

The mechanically crushed concrete, for instance from a demolition of the old, worn out objects, is used as a recycling aggregate. The artificial aggregates for concrete, resulting from the sintering of the industrial ashes, or various furnace and metallurgical slags are used. Utilization of some hazardous wastes is also possible after suitable processing [5,6,7,8]. The production of cement consumes significant amount of the wastes, used as alternative fuels for cement kilns [9,10,11]. In Poland, about 10% of the municipal wastes is utilized this way. Production of cement allows to utilize some by-products of the industrial combustion processes as a primary components of the cement. They are, among others, the fly ashes, blast-furnace slags or silica fume [12,13]. These materials, however, need to full fill the requirements concerning the chemical composition, defined in the European or national standards. Various mineral flours, for example resulting from the processing of the natural aggregates, may be used as secondary components—up to 5% of the cement mass [14].

The other possibility of utilization of the wastes in the cement composites is their use as additions for concrete, i.e., in quantities exceeding 5% of the cement mass. According to EN 206 [15], the additions for concrete can be of two types: they are almost inert to the components of the cement paste or have pozzolanic ability or latent hydraulic ability (the so-called active additions). The active additions have much more potential in the range of their use as substitutes for the part of cement, maintaining the similar or even better performance of the composite [16]. The process of combustion of the coal and lignite in the boilers with the dust furnaces is accompanied by production of the ferromagnetic compounds, contained in the fly ashes and slags. As it was demonstrated in the research [17], the highest amounts of the magnetic iron compounds occur in the silica fly ashes, the smaller amounts in the alumina fly on ashes, and the smallest in the calcium fly ashes. When selection of the raw material for extracting the metal concentrates from the combustion by-products is carried out, the amount of the compounds of the given metal in the coal is not always the most important factor. The real crucial factor is the structure of the present compounds and the technological sequence. The significantly richer metal concentrates can be often obtained from the raw material containing lesser volumes of the specific compounds. The greatest recovery of the metals is achieved when the methods of chemical de-sulfurization and de-mineralization, as well as the physical methods, are employed [18]. The research conducted in Poland allowed to implement the technology of the production of the iron oxides concentrates. The method of extracting the ferromagnetic fraction from the fly ashes was developed, and the conducted implementations made possible to produce the magnetite in the volume up to 20,000 tons per year [19]. The largest amounts of the magnetite concentrate were used in Poland in the coal mining for preparing the heavy liquid for the output enrichment [17].

The other way of utilization of the iron oxides concentrate and post-flotation wastes is their use for the production of cement cement-based building materials. The cement industry is facing the challenge connected with insufficient supply of the raw materials and necessity of environmental protection. Therefore, for some time it searches for alternative raw materials for the production of Portland clinker. Various industrial wastes are used for this aim, such as steel slag, ashes from the waste muds and ceramic wastes [20,21,22]. Because of the high content of the iron, the magnetite concentrate and the solid tailings resulting from the iron ore enrichment (IOT) are used as an iron correcting material during production of the Portland clinker [23]. The use of these materials, however, is rather low. The waste concentrates of the iron oxides are used in the cement-based building materials as a substitute of the natural aggregate. They are often used for the production of the heavy-weight concretes. Mironovs et al. [24] used the wastes of the powdered iron for the production of the heavy-weight concretes intended for the use as counterweights. The tests have demonstrated that the introduction of the waste iron powders significantly increased the density and Young modulus of these concretes. The partial replacement of the sand (up to 35%) in the concrete allowed to improve the flexural and compressive strength [25,26].

The research [27] concerned the use of the IOT as: addition to the cement mortar (up to 20% of the sand), substitute for the sand in the mortar (20%, 60% and 100%) and substitute of the cement in the mortar (10%, 20% and 30% of the cement mass). The investigation has demonstrated that the addition of 20% of IOT to the cement mortar only slightly decreased the final mechanical properties of the composites. Replacement of the sand by 20, 60 and 100% of IOT was significantly more favorable and improved the strength. A decrease of the strength was observed in the case of the magnetite powder that was used as a substitute of cement in the building composites [28].

The cement composites containing the addition of the iron oxides concentrates (IOC) can be used in the production of the biological shields against the ionizing radiation for the hospitals and radiotherapy centers. The use of the natural aggregates with high specific weight increases significantly the cost of production of the shielding concretes. Therefore, the powdered or coarse additions containing the heavy metals are increasingly used for manufacturing such concretes [29]. Commonly known is the use of the lead powders [30] and aggregates [31], but due to the potential toxicity [32] the use of lead in the shielding concretes has been limited. For this reason, such wastes as the IOT, iron oxides concentrates obtained from the combustion of the minerals and the wastes from the processes of mining and crushing the igneous rocks are increasingly used in the shielding concretes [33,34]. The research [35] has shown that the addition of the magnetite powder up to 20% of the cement mass can be successfully applied to the concrete, this way increasing its linear damping ratio. Addition of 30% of IOT applied as a fine aggregate for the cement mortars significantly improved their shielding performance [36]. The researches including [34,37,38,39] have confirmed that the use of the larger amount (above 30% of the cement mass) of the iron oxides concentrate and other waste powders containing the metal oxides worsens significantly the consistency of the mortars and the mechanical performance of the hardened cement composites.

The cement-based building materials should demonstrate, besides the load-bearing capacity, also the proper thermal resistance. Such design of the materials is connected with the fire requirements for the building structures and materials [40]. Selection of the suitable aggregate plays a key role in ensuring the satisfactory thermal resistance of cement mortars and concretes, since the aggregate makes up from 60% to 80% of the composite’s volume [40,41]. The aggregate resistant to the high temperature should demonstrate low loss of weight and low thermal expansion coefficient [41,42]. Most of the aggregates used for production of cement-based building materials remain stable at the temperature of 500–600 °C. The silica aggregates (i.e., quartzite, granite) show poorer thermal resistance than the carbonate aggregates (e.g., limestone or dolomite) [43,44]. This results from the higher thermal expansion of the silica aggregates and phase transition of the quartz crystals that takes place at 573 °C, increasing the volume of the material [45]. The carbonate aggregate are destroyed at the temperature of 700 °C to 900 °C due to the decomposition of the calcium carbonate; CaCO_3_ is decomposing into (lime) and CO_2_ (carbon dioxide) [41,46,47]. The weak link in the range of the thermal resistance of the cement composites is a cement paste. The main components of the hydrated cement paste are calcium silicate gel, C–S–H (the main binding phase, constituting 50–60% of the paste volume) and calcium hydroxide, CH (20–25% of the volume) [44]. The process of degradation of the cement paste starts from the very beginning of heating, although up to the temperature of 300 °C the changes are considered slight and reversible (the so-called re-hydration). The thermal resistance of the cement paste is affected by many factors, of which the most important are water-to-cement ratio (w/c), calcium oxide-to-silicon dioxide ratio (C/S) and the amount of CH. The paste with low value of C/S and low CH content enables to manufacture the heat resistant cement composite [43,47]. Decomposition of C–S–H phase starts from the very beginning of the heating process, which leads to the increase of the total porosity of the paste and changes in the pores structure [41,47]. Yet up to 300 °C no drastic change in the porosity is observed. After exceeding the temperature of 300 °C the porosity of the paste increases significantly. The temperature 600–800 °C is considered the limit temperature causing the lowering of the load-bearing capacity of the cement composites due to the physico-chemical changes in the cement paste. The intensive cracking occurs in the aggregate-paste contact zone [47]. Both mortars and concretes at these temperature remain about 20% of their load-bearing capacity. The loss of chemically bound water and total decomposition of C-S-H phase takes place at the temperature of 800–1000 °C [48]. The loss of the entire strength of the cement composites occurs within this range of the temperature [49].

According to the authors’ knowledge, the investigations of the mechanical performance of the cement-based building composites containing IOC have so far been carried out at the temperature of 20–25 °C. The authors have tested the mechanical properties of the cement mortars with the addition of the IOC heated at the temperature 300, 450 and 600 °C. The IOC was used as a substitute of the fine aggregate in the amount of 10, 20 and 30% (by mass). The purpose of the studies was to show the other possibilities of using the waste IOC in the cement-based building materials. The obtained results have confirmed the possibility of using the IOC as a substitute for the natural aggregate in the building materials with the improved resistance to the high temperature up to 450 °C.

## 2. Materials and Test Methods

### 2.1. Materials

**Figure 1:**
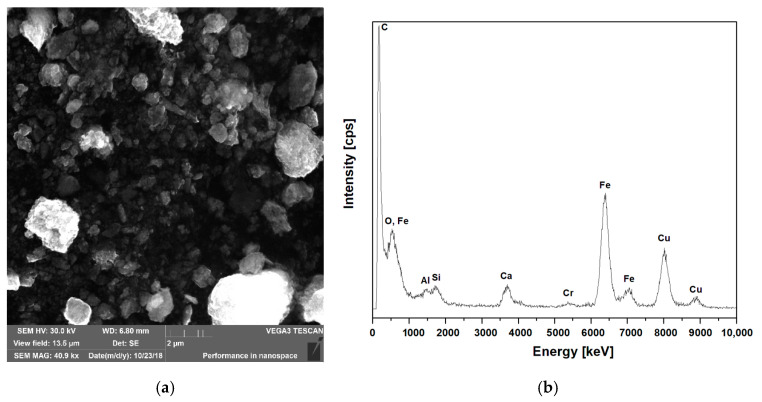
SEM of iron oxides concentrate and EDS analysis. (**a**) SEM image of IOC; (**b**) EDS analysis.

The cement pastes and mortars were prepared using Portland cement CEM I 42.5R (Górażdże Cement S.A., Górażdże, Poland), with the specific surface area about 370 m^2^/kg and the specific weight 3120 kg/m^3^. The iron oxides concentrate (IOC) used in the investigation, with the average specific weight 4100 kg/m^3^, was a waste obtained during enrichment of the iron ore. To determine the chemical composition of the IOC, a wave dispersion X-ray fluorescence spectrophotometer (WDXRF) by Malvern Panalytical (Malvern, UK) was used. The scanning electron microscopy (SEM) images of the raw IOC are shown in the Figure 1. There were two types of particles: the irregularly shaped agglomerates and the porous structures. The average particle size was between 0.125 mm and 0.25 mm. Energy-dispersive X-ray spectroscopy (EDS) analysis confirms the presence of significant amounts of iron (Figure 1) SEM and EDS analyzes were performed using a VEGA3 TESCAN scanning microscope (TESCAN, Brno, Czech Republic). The chemical composition of the IOC is presented in the Table 1 and the grain size distribution of the concentrate is given in the Table 2. The river sand with the specific weight 2650 kg/m^3^ was used as a fine aggregate; the grain size distribution of the sand is presented in the Table 2. The mortars were prepared using superplasticizer containing the polynaphtalene condensates and melamine (BASF Polska, Myślenice, Poland) with the density 1140 kg/m^3^. Four cement mortars were designed with the constant water to cement ratio, w/c = 0.5. The reference mortar (M0) without addition of IOC was prepared without superplasticizer. The mortars denoted as M10, M20 and M30 contained IOC as the substitute for the aggregate in the amount 10%, 20% and 30% of the aggregate mass.

All mortars containing the addition of IOC were designed in such a way that their consistence was close to that of the reference mortar and was within the range 150–160 mm (determination of consistence by the method of the flow table according to BS EN 1015-3). The compositions of the tested mortars are presented in the Table 3.

The cement pastes for testing the heat of hydration were designed with the constant water to binder ratio 0.5; the binder consists of cement and IOC. The tests were carried out on the paste without the concentrate P0 (reference paste) and three pastes in which the cement was partially replaced with IOC in the amount 10%, 20% and 30%, denoted as P10, P20 and P30, respectively. The compositions of the tested pastes are presented in the Table 4.

### 2.2. Test Methods

Effect of IOC on the cement hydration was investigated using isothermal calorimeter TAM Air at the temperature 20 ± 2 °C. The tests were performed on the cement pastes of the compositions given in the Table 4. The mass of every paste specimen was 45 g, including 15 g of the water and 30 g of the binder (w/b = 0.5). The other tests were carried out on the fresh and hardened cement mortars with the IOC as the substitute for the aggregate. Because of the use of a superplasticizer in the amount proportional to the IOC content, the mortars gained the desirable consistence (flow 150–160 mm) and showed no segregation during mixing.

**Figure 2:**
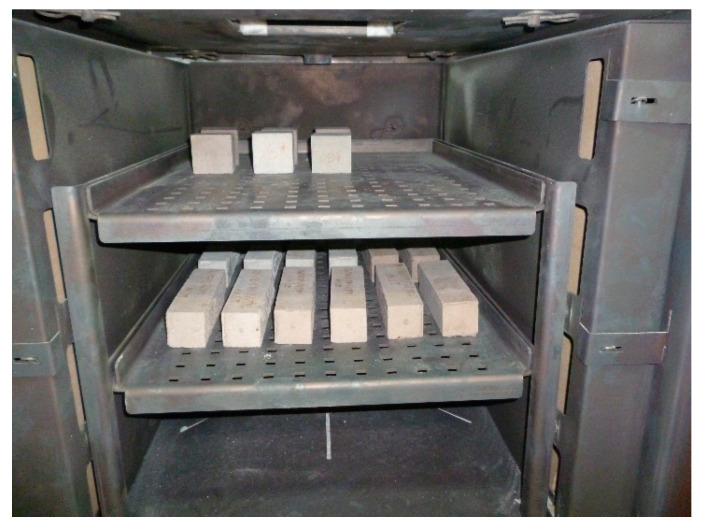
Samples in the furnace.

The flexural and compressive strength of the mortars was determined according to the Standard BS-EN 196-1 [50]. The mortars specimens were prepared in the form of the beams of the sizes 40 mm × 40 mm × 160 mm. The specimens after demoulding were stored in the climatic chamber at the relative humidity RH ≥ 95% and the temperature 20 ± 2 °C for 28 days until the testing. The specimens intended for the heating (at 300 °C, 450 °C, 600 °C and 800 °C), after curing for 28 days, were weighed and dried to the constant mass in the laboratory dryer at the temperature 105 °C. After drying the specimens were weighed again to determine the density in the dried state and then heated at the given temperatures. The heating was carried out in the chamber furnace PP 140/85 provided with temperature controlling device Ht Industry. The specimens were placed on the shelves in the furnace without contacting each other and without touching the heating elements (Figure 2). The heating procedure followed the RILEM TC 200-HTC recommendation [51]. In the first stage the temperature increases at the rate 1 °C/min. The maximum temperature was maintained for 1 h. The cooling runs at the rate 1 °C/min. The temperature in the furnace was controlled using the temperature control device and the thermal probe placed inside the furnace. The specimens were removed from the furnace when the temperature in the furnace was 50 °C; the previous studies done by the Authors have demonstrated that this ensures the repeatability of the conditions of heating and cooling the specimens.

After heating, the specimens were visually inspected and weighed again for determination of the mass loss. Then the specimens were cooled down at the temperature 20 ± 5 °C and relative humidity 50 ± 5%. After cooling, the specimens were tested mechanically for determination of the flexural and compressive strength. The obtained results of the strength testing of the mortars M10, M20 and M30 were compared to the strength of the mortars cured for 28 days in the conditions described in the standard [52] and not subjected to the heating (the mortars M0). Three specimens were prepared of each mortar for heating at the given temperature.

The cube specimens of the sizes 50 mm× 50 mm× 50 mm were prepared for testing the thermal conductivity of the mortars. The measurement of the thermal conductivity coefficient was carried out after 28 days of curing the specimens, after drying the specimens to the constant mass and polishing the surface. The tests were conducted by non-stationary method (the hot-disk method) using the ISOMET 2104 equipment (Applied Precision Inc., Vaughan, ON, Canada) with the measuring heads intended for the materials with the thermal conductivity in the range 0.3–2.0 W/(m·K) and 2.0–6.0 W/(m·K).

After 2 and 28 days of curing the compressive and flexural strength of the specimens were tested in accordance with BS-EN 196-1 [52].

## 3. Test results and Discussion

### 3.1. Heat of Cement Hydration

**Figure 3:**
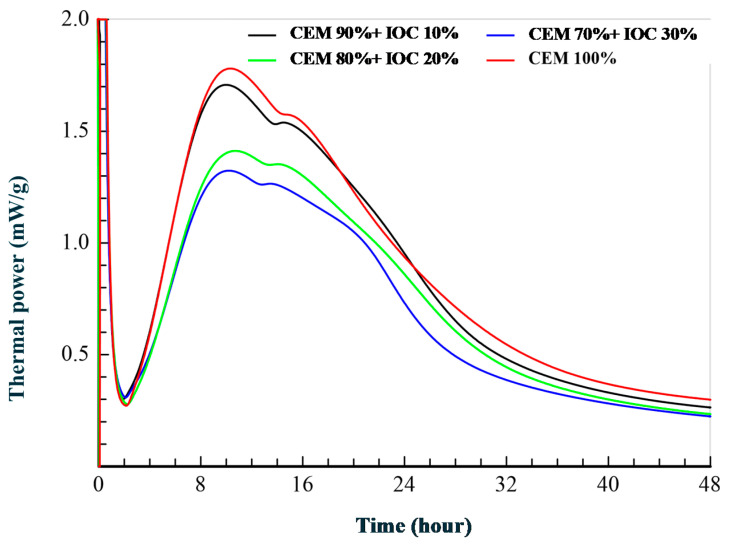
Heat flow calorimetry of the cement with different dosages of IOC.

**Figure 4:**
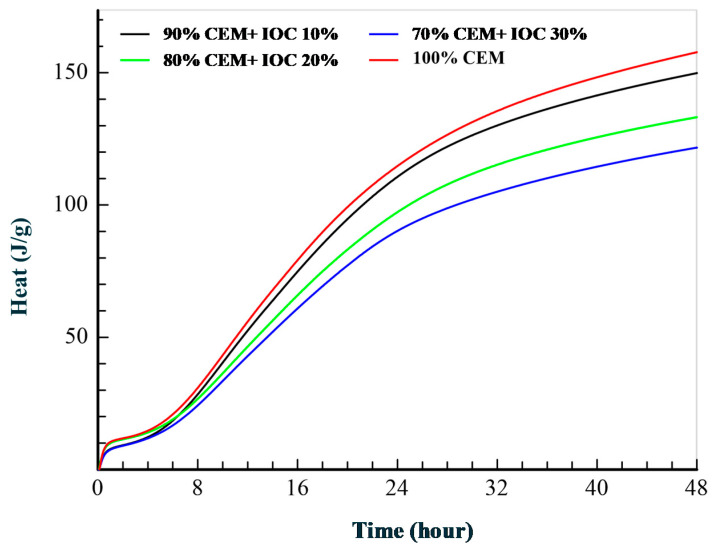
Cumulative hydration heat curves.

The curves illustrating the rate of the heat emission by the cement pastes containing 10, 20 and 30% of the magnetite powders and the reference paste are presented in the Figure 3. The cumulative heat emission is presented in the Figure 4.

The inflection on the sloping part of the curve is visible for all pastes in the main peak of heat emission by cement itself. This inflection is connected with the ettringite formation. The maximum values of the rate of heat emission decreases with increasing content of IOC in the pastes. The larger content of the IOC in the paste (the specimens with 30% of IOC) caused atypical course of diminishing of the heat emission rate, what is clearly visible on the curve. This points to the slight acceleration of the cement hydration, which can be caused by the gradual releasing the adsorbed water by the concentrate particles. As it was demonstrated in the research [53], such shape of the curve is a result of the “filler” effect consisting in the fact that addition of any fine-grained material accelerates the alite reaction rate. Increased reaction of alite means that more sulfate is absorbed by the C–S–H during the main heat peak and therefore the sulfate depletion peak occurs earlier. The research [54] shows that the effectiveness of this process does not depend on whether the addition is chemically active or inert in relation to the clinker phases of the cement. The acceleration of the cement hydration is a result of the physical action of the fine-grained addition, what is visible in the case of the paste P30, containing the largest amount of the fine fraction.

### 3.2. Density and Thermal Conductivity

**Figure 5:**
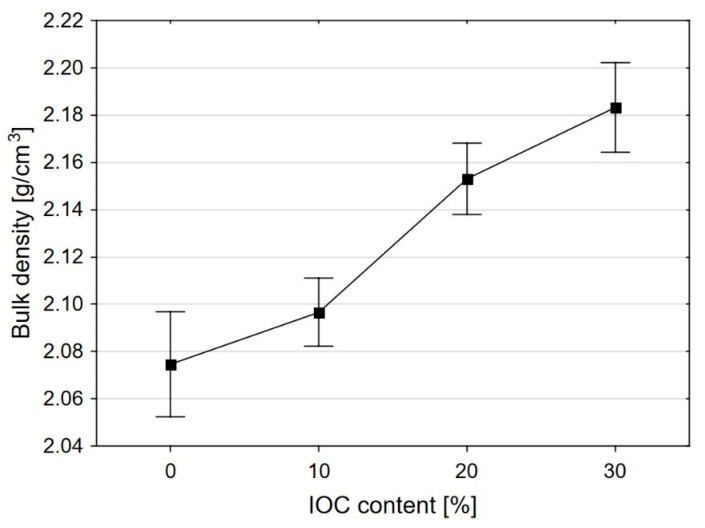
Change of the bulk density of mortars as a function of IOC share in the aggregate.

The Figure 5 presents the dependence of the apparent density and thermal conductivity of the mortars specimens on the IOC content in relation to the fine aggregate. The addition of IOC increases the apparent density as compared to the reference mortar. This increase is approximately monotonic, which is connected, basically, with the presence of the additional amount of the superplasticizer in the mortar. The average apparent densities of the tested mortars in the natural state and after drying at 105 °C are presented in the Table 5.

**Figure 6:**
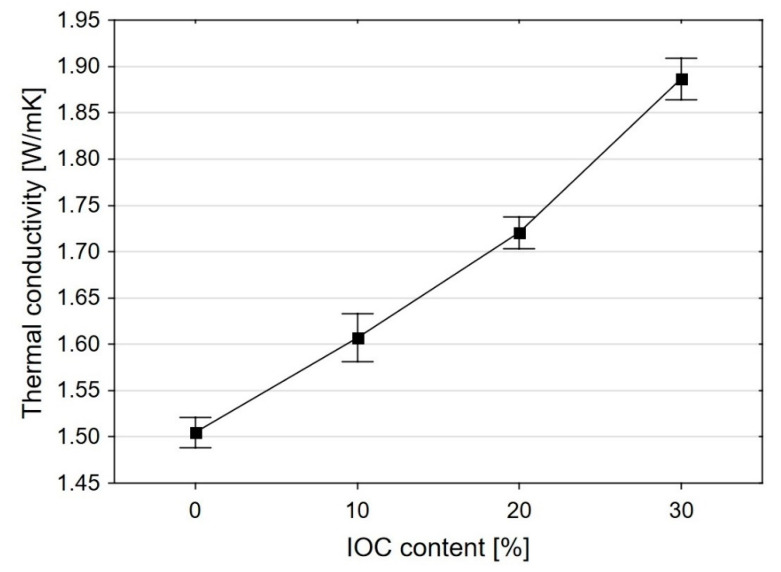
Change of the thermal conductivity of the mortars as the function of the IOC share in the aggregate.

The thermal conductivity coefficient of the mortars, l, strongly depends on the share of IOC in the fine aggregate (Figure 6). Replacement of 10% of the sand volume by the IOC has caused an increase of the thermal conductivity on average by 28%. The thermal conductivity of the mortar M0 was on average 0.92 W/mK, while for the mortar M10 this value was 1.18 W/mK. In the case of the mortars M20 and M30 increase of the thermal conductivity in relation to the reference mortar M0 is not so big. This results from increasing content of the superplasticizer in the mortar. The research [55] has confirmed that addition of the IOC as the substitute for the aggregate leads to the significant increase of the thermal conductivity of the mortars. The coefficient l within the range from 1.5 to 1.95 W/mK (at the temperature of 25 °C) has been obtained for the mortars containing the IOC as the aggregate at the w/c = 0.5 [55].

### 3.3. Properties of the Hardened Mortars at the Temperature of 20 °C

The cement mortars specimens were tested after 28 days of curing in the controlled environment at the temperature 20 ± 2 °C. The flexural strength was measured on 6 specimens of each mortar and the compressive strength was measured on 3 specimens of each mortar. The results of testing are presented in the Table 5.

In the case of the mortars containing 10% and 20% of IOC (the mortars M10 and M20) a slight increase of the flexural strength in relation to the reference mortar was observed—by 2.2% and 5%, respectively. The compressive strength of the mortars M10 and M20 has increased in relation to the reference mortar by 8.9% and 10%. For the mortar M30 an increase of the flexural and compressive strength was also observed (by 2.7% and 8.8%, respectively), but the downfall tendency is visible as compared to the results obtained for the mortar M20. A decrease of the strength of the mortar M30 as compared to the mortar M20 is a result of an increase of the share of the fine fraction 0.25 to 0.125 in the entire aggregate, which will worsen the compressive strength with the further growth of the IOC content. Nevertheless, the substitution of 30% of the sand with the IOC allows to improve slightly the strength parameters of the mortars and keep the good workability (the superplasticizer was used in the amount of 2.5% of the cement mass). The similar results were reported in [38]. Worsening of the mechanical performance of the mortars was observed at 18% share of IOC in the aggregate, however no superplasticizer was used. An increase of the strength of the tested mortars is a result of the fact that the IOC acts as a filler, sealing the structure of the mortars. The dusty fractions of the IOC could act as crystallization nuclei for C–S–H phase, causing compaction of the composite structure and increase of the strength [13,49]. The SEM analysis in this range is necessary in the future. Investigation of the pastes hydration heat has confirmed that the addition of IOC can be used as a filling aggregate (addition of type I according to the European Standard EN 206 [15]) in the cement composites.

### 3.4. Properties of Hardened Mortars after Exposure to High Temperature

Degradation of the cement matrix starts from the very beginning of heating the cement composites. The changes in the composite observed when heating up to 300 °C, however, are relatively small and can be reversible (the re-hydration is possible) [56,57]. The capillary water is totally lost at the temperature of 400 °C [41]. The biggest changes of the porosity of cement matrix connecting the aggregate grains are observed in the range of temperature 300–600 °C [48]. Within this range of temperature the hydrated calcium silicates C–S–H are dehydrated. Dehydroxylation of the portlandite Ca(OH)_2_ is completed at 600–700 °C [48]. When temperature reaches 400 °C the microcracks in the cement matrix and aggregate start to propagate, and their intensity grows with the rising temperature [58,59,60,61,62,63]. The cement composites are losing their load bearing capacity at 800–1000 °C [64].

On the basis of the literature review, the range of temperature from 300 °C to 800 °C has been accepted in the tests. The mortars specimens in the form of the beams with the sizes 40 mm× 40 mm× 160 mm were heated in the electric furnace according to the procedure described in the Section 2.2. The specimens heated at the temperature of 800 °C were destroyed. After removing from the furnace, the specimens crumbled and no further tests were possible to carried out. The reason of the destruction of the specimens heated at 800 °C, as it was shown in the research [48], is breaking of the aggregate-matrix bonds.

**Figure 7:**
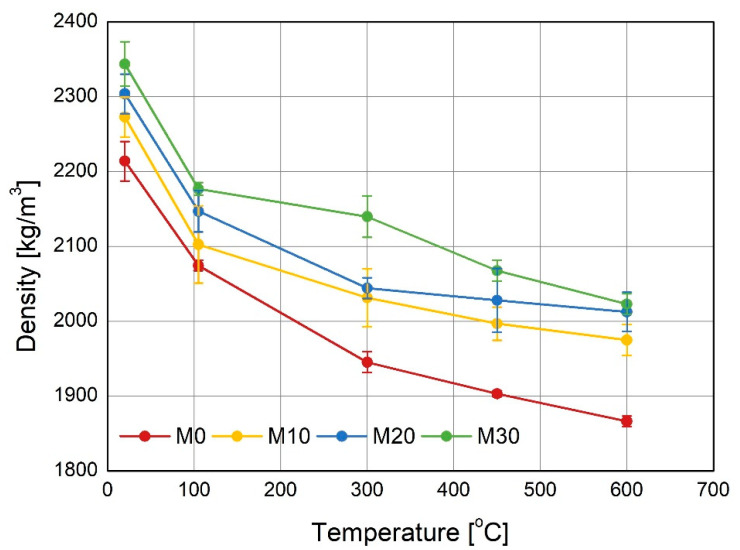
Change of the specimens density as a function of the heating temperature.

The changes in the density of the mortars specimens during heating at the high temperature are presented in the Figure 7. The clear impact of the IOC content in the aggregate on the density of the tested mortars is visible. The aggregate containing the IOC had finer grains distribution (see Table 2) and thus increased water demand. However, due to the higher density of the IOC and the use of superplasticizer, the initial densities of the mortars M10, M20 and M30 were higher. During the first stage of drying (at 105 °C) the free water, absorbed by the specimens when cured, has evaporated. In the consecutive stages of heating the fall of the density of the specimens containing the IOC is less rapid than that of the reference mortar M0. The decisive effect on the less falls of the densities of the mortars with IOC has the increased thermal conductivity of the concentrate, leading to the less cracking of the specimens during heating. This, in turn, causes the increase of the flexural and compressive residual strength, shown in the following figures.

**Figure 8:**
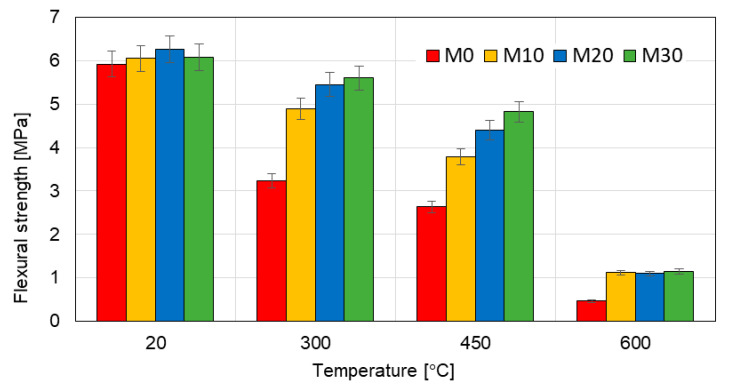
Flexural strength of the mortars heated at various temperature.

**Figure 9:**
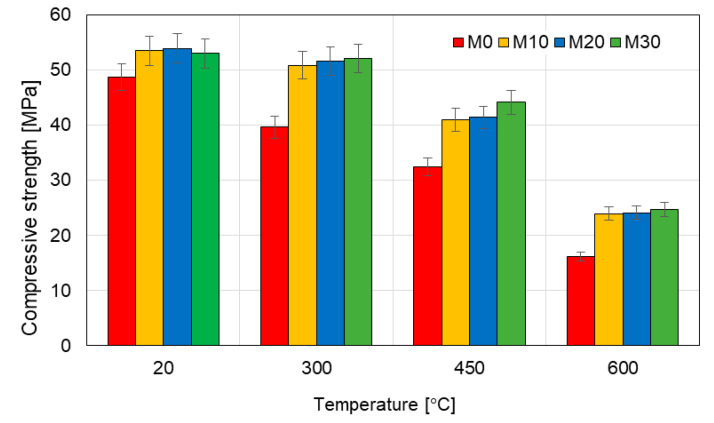
Compressive strength of the mortars heated at various temperature.

The results of the tests of the flexural and compressive strength are presented in the Table 6 and in the Figure 8 and Figure 9. The flexural strength of the mortars modified with the IOC at the temperature of 20 °C is quite similar (an increase by 5% as compared to the reference mortar M0), but at 300 °C the differences are significant. The flexural strength of the mortars M10, M20 and M30 at the temperature of 300 °C is higher as compared to the reference mortar M0 by 51%, 68% and 72%, respectively. The flexural strength of the mortars M10, M20 and M30 at the temperature of 450 °C is higher as compared to the reference mortar M0 by 43%, 66% and 83%, respectively.

At the temperature of 600 °C the strength of all tested mortars diminished, but the flexural strength of the mortars containing IOC was still more than twice higher than that of the reference mortar M0. Similar, however not so big, differences were observed in the case of the compressive strength. Here also the mortars with the addition of the IOC have demonstrated much better resistance to the high temperature than the reference mortar M0 (Figure 8).

The falls of the compressive strength of the mortars M10, M20 and M30 at the temperature of 300 °C did not exceed 5% as compared to the strength at the temperature of 20 °C, i.e., were within the measurement error. The compressive strength of the specimens with the IOC heated at the temperature of 300 °C was on average by 30% higher than in the case of the reference mortar M0. At the temperature of 450 °C the compressive strength of the mortars M10, M20 and M30 was higher as compared to the reference mortar M0 by 26%, 27% and 36%, respectively. The compressive strength of the specimens with the IOC heated at the temperature of 600 °C was similar (about 24 MPa) and on average by 48% higher than the compressive strength of the reference mortar, which was 16 MPa.

**Figure 10:**
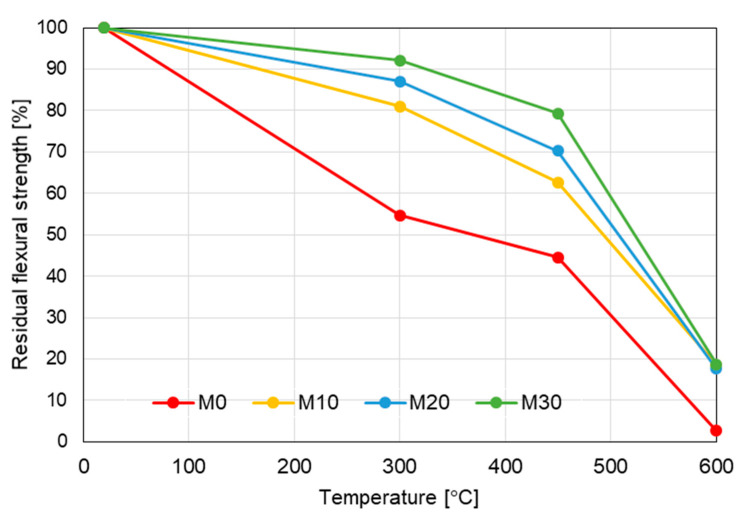
Evolution of residual flexural strength with temperature.

The Figure 10 presents the residual flexural strength of the tested mortars specimens. The positive effect of the addition of IOC is clearly visible. At the temperature of 300 °C the reference mortar (M0) kept slightly above 50% of its initial flexural strength (54.74%), while the flexural strength of the mortars M10, M20 and M30 was 81%, 87% and 92%, respectively, of their strength at 20 °C.

**Figure 11:**
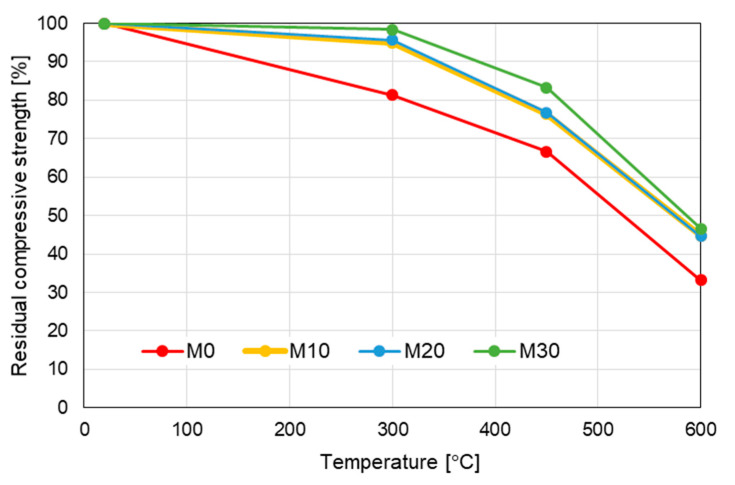
Evolution of residual compressive strength with temperature.

The similar relations were obtained in the case of the residual compressive strength of the mortars heated at the high temperature (Figure 11).

Similarly as in the case of the flexural strength, it is visible that the addition of IOC improves the mechanical performance at the high temperature. At 300 °C the mortars modified with the IOC maintain from 95% (the mortars M10 and M20) to 98% (the mortar M30) of their initial strength, while the reference mortar M0 reaches only 80% of its initial compressive strength. By analogy, in the case of heating up at the temperature of 450 °C the mortars with the IOC keep from 76% to 83% of their initial strength, while the mortars M0 only 66%. At the temperature of 600 °C all mortars with the IOC have similar residual strength about 45%, while the mortar M0 only 33%.

**Figure 12:**
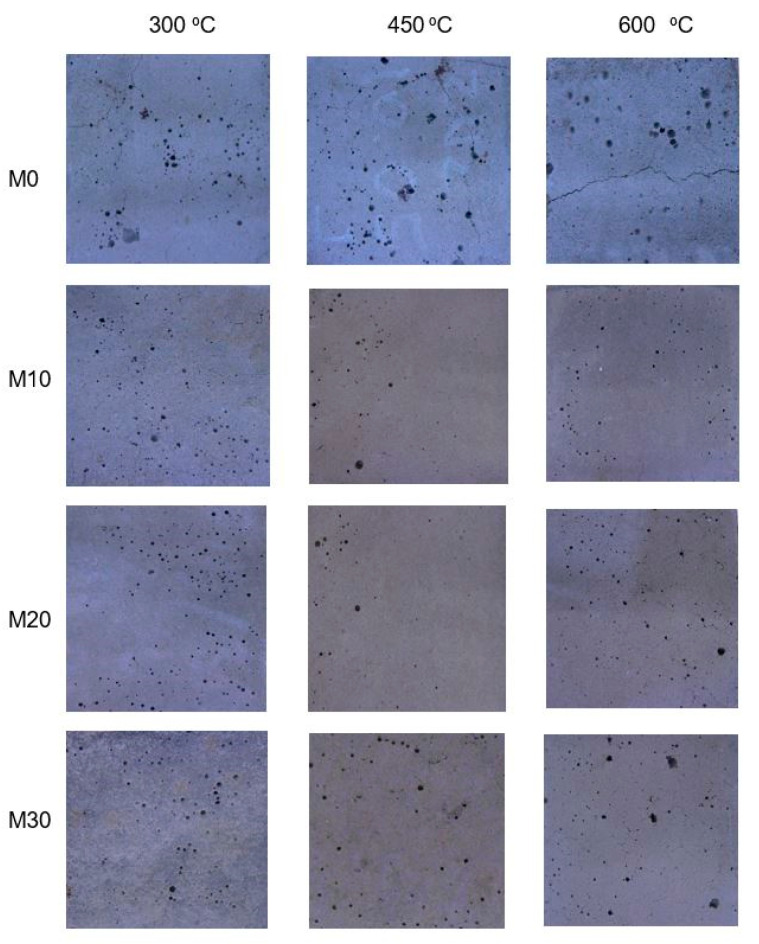
Pictures of four cement mortars heated at 300, 450 and 600 °C.

As follows from the analyses presented above, the substitution of the part of the aggregate with the IOC has caused a significant improvement of the mechanical performance of the cement mortars at the high temperature. Improvement of the mechanical performance of the mortars, also at the high temperature, results from the role of the filler played by the used IOC. This, in turn, is transferred into the limitation of the composite’s porosity and compaction of its structure. This improvement is also connected with the higher thermal conductivity of the concentrate used [65], which limits the cracks propagation at the temperature 300–450 °C. This has been confirmed by the appearance of the mortars specimens surfaces after heating (Figure 12). In the case of the mortar M0 a number of cracks occurs on the specimens surfaces already at the temperature of 300 °C. At 600 °C these cracks merge into one running along the specimen axis. The cracking occurs over the entire height of the specimen, leading to the minimum compressive and flexural strength. At the temperature of 300 °C the single cracks are visible on the mortar M10 specimens’ surfaces, while the surfaces of the mortars M20 and M30 specimens remain uncracked. At the temperature of 450 °C the surfaces of the specimens of the mortars containing IOC are covered with the fine radial cracks, running from the large air voids. Their width, however, is even twice as small as in the case of the mortar M0. At the temperature of 600 °C the numerous cracks form the closed grid on covering the entire surface. The largest cracks occur around the macropores, which were formed as a result of the air-entrainment of the mortar during preparation of the specimens. The higher was the content of the IOC in the mortar, the higher was porosity of the specimens, visible on their surfaces. The improvement of the mechanical performance of the mortars containing the IOC at the high temperature can also be explained by the stronger bonds in the aggregate-paste contact zone [46,66], which become apparent in the running of the cracks of the heated specimens. The physico-chemical properties of the IOC also affect positively the properties of the modified mortars. The high content of Fe_2_O_3_ in the IOC (more than 69%) causes that the damages of the aggregate and the matrix at the elevated temperature occur much later than in the case of the reference mortar. This is visible both in the cracking image on the surface as well as in the values of the residual strength (Figure 9 and Figure 10). The reason for that is the performance of Fe_2_O_3_ at the high temperature. The phase transition of Fe_2_O_3_ takes place not earlier than at the temperature of 723 °C, and Fe_2_O_3_ melting temperature is much higher, ranging from 1538 °C to 1565 °C [66]. The improvement of the physico-mechanical properties at the high temperature was possibly caused also by a presence of the reactive SiO_2_ in the IOC [49]. The physico-mechanical properties of the cement composites containing the IOC, also at the high temperature, are significantly connected with the chemical composition of the concentrate. The chemical composition of the IOC is, in turn, closely related to the method of the iron ore enrichment [67,68]. The high content of SiO_2_ and Al_2_O_3_ in the IOC makes possible to use the waste IOC as an admixture to the cement. The majority of the published research [23,25,26,55], however, point to the use of the IOC as the substituent for the fine aggregate in the cement composites. The carried out tests have shown that the IOC can be used for the improvement of the physico-mechanical properties of the cement-based building composites at the high temperature.

## 4. Conclusions

Three cement mortars were tested, in which a part of the aggregate was replaced with the waste iron oxides concentrate (IOC) in the amount of 10%, 20% and 30% of the aggregate mass. The obtained results were compared with those obtained for the reference mortar without addition of the IOC. The following conclusions have been formulated:

1. The addition of the IOC does not affect chemically the cement hydration. When used in the large amount like 30% of the aggregate mass, however, can cause some acceleration of the cement hydration as a result of the physical impact of the fine-grained addition.
2. All mortars containing the IOC showed an increased compressive strength after 28 days of curing, as compared to the reference mortar M0 (without the IOC). This is an effect of the better filling the cement matrix with the concentrate finer and harder than the natural sand.
3. Due to the higher specific weight of the IOC in relation to the natural aggregate, the apparent density of the mortars increases with the growing content of the IOC addition. The specimens of the mortars containing 10%, 20% and 30% of the IOC have demonstrated the higher thermal conductivity than the reference mortar, in proportion to the IOC content. This is explained by the high thermal conductivity of the IOC itself as well as the low absorbability (low open porosity).
4. The addition of the IOC improves significantly the residual strength of the cement mortars at the high temperature. The use of only 10% of the IOC addition makes possible to keep 95% of the initial compressive strength of the mortar at the temperature of 300 °C. The mortars containing the IOC maintain from 76% to 83% of their initial compressive strength at 450 °C, while the reference mortar only 66%. This is caused by acting of the IOC as a filler, which leads to the compaction of the structure of the cement matrix. High content of Fe_2_O_3_ in the IOC causes the limitation of the composite cracking due to the high thermal conductivity and phase transition of iron oxide at the temperature of 723 °C. The key issue for the desirable improvement of the mechanical performance of the mortars contained IOC is ensuring the proper workability of the mortars for reducing their porosity. This can be obtained by using the increased amount of a superplasticizer.
5. Due to the higher apparent density and improved flexural and compressive strength of the mortars with the addition of the IOC at the high temperature, the IOC can be used as a substitute for the fine aggregate in the production of the mortars resistant to the high temperature (up to 450 °C), as well as to produce the heavy-weight mortars and concretes for the structures exposed to ionizing radiation.

## Figures and Tables

**Table 1:** Chemical composition of iron oxide concentrate (IOC) [wt.%].

**Compound Formula**	**Fe_2_O_3_**	**SiO_2_**	**Al_2_O_3_**	**CaO**	**MgO**	**Cr_2_O_3_**	**NiO**	**SO_3_**	**MnO**
Concentration	69.451	11.425	5.643	4.892	3.404	3.364	0.452	0.439	0.392
**Compound Formula**	**TiO_2_**	**Na_2_O**	**P_2_O_5_**	**K_2_O**	**V_2_O_5_**	**BaO**	**ZnO**	**CuO**	**Cl**
Concentration	0.121	0.120	0.069	0.061	0.057	0.041	0.031	0.026	0.012

**Table 2:** Grain composition of the river sand and IOC used for the preparation of the mortar specimens.

Sieve Size [mm]	Passing (%)					
	2	1	0.5	0.25	0.125	0.0625
River sand	100	86.6	45	8.1	0.6	0.1
IOC	100	100	98.9	47.65	1.01	0.0

**Table 3:** Mix proportions of cement mortars [kg/m^3^] and flow of the fresh mortars [mm].

Mortar Code	Cement	Water	Fine Aggregate	IOC	IOC (% of Aggregate Mass)	Super-Plasticizer	Flow [mm]
M0	512	256	1535	0	0	0	150
M10	512	256	1381	154	10	5.85	158
M20	512	256	1228	307	20	6.75	157
M30	512	256	1074	461	30	9.90	158

**Table 4:** Pastes compositions.

Paste Designation	Components (g)			Ratio	
	Cement	IOC	Water	Water/Cement w/c	Water/Binder w/b
P0	30	0	15	0.50	0.5
P10	27	3	15	0.56	0.5
P20	24	6	15	0.63	0.5
P30	21	9	15	0.71	0.5

**Table 5:** Properties of 28-day-old hardened mortars.

Mortar Code	M0	M10	M20	M30
Flexural strength (MPa)	5.92 ± 0.30	6.05 ± 0.30	6.26 ± 0.31	6.08 ± 0.30
Compressive strength (N/mm^2^)	48.68 ± 2.43	53.05 ± 2.65	53.90 ± 2.70	52.98 ± 2.65
Density (kg/m^3^)	2214 ± 26	2273 ± 27	2304 ± 26	2344 ± 30
Density after drying at 105 °C (kg/m^3^)	2075 ± 7	2101 ± 52	2153 ± 28	2180 ± 8

**Table 6:** Flexural and compressive strength of the cement mortars heated at the high temperature.

Mortar Code	Flexural Strength [MPa]				Compressive Strength [MPa]			
	20 °C	300 °C	450 °C	600 °C	20 °C	300 °C	450 °C	600 °C
M0	5.92	3.24	2.64	0.46	48.68	39.62	32.43	16.17
M10	6.05	4.90	3.79	1.12	53.50	50.85	40.97	23.92
M20	6.26	5.45	4.40	1.10	53.90	51.56	41.39	24.08
M30	6.08	5.60	4.82	1.14	52.98	52.11	44.12	24.69

## Data Availability

Data sharing is not applicable to this article.
